# Regional physiology of ARDS

**DOI:** 10.1186/s13054-017-1905-9

**Published:** 2017-12-28

**Authors:** Luciano Gattinoni, Tommaso Tonetti, Michael Quintel

**Affiliations:** Department of Anesthesiology, Emergency and Intensive Care Medicine, University of Göttingen, Göttingen, Germany

## Abstract

The acute respiratory distress (ARDS) lung is usually characterized by a high degree of inhomogeneity. Indeed, the same lung may show a wide spectrum of aeration alterations, ranging from completely gasless regions, up to hyperinflated areas. This inhomogeneity is normally caused by the presence of lung edema and/or anatomical variations, and is deeply influenced by the gravitational forces.

For any given airway pressure generated by the ventilator, the pressure acting directly on the lung (i.e., the transpulmonary pressure or lung stress) is determined by two main factors: 1) the ratio between lung elastance and the total elastance of the respiratory system (which has been shown to vary widely in ARDS patients, between 0.2 and 0.8); and 2) the lung size. In severe ARDS, the ventilatable parenchyma is strongly reduced in size (‘baby lung’); its resting volume could be as low as 300 mL, and the total inspiratory capacity could be reached with a tidal volume of 750–900 mL, thus generating lethal stress and strain in the lung. Although this is possible in theory, it does not explain the occurrence of ventilator-induced lung injury (VILI) in lungs ventilated with much lower tidal volumes. In fact, the ARDS lung contains areas acting as local stress multipliers and they could multiply the stress by a factor ~ 2, meaning that in those regions the transpulmonary pressure could be double that present in other parts of the same lung. These ‘stress raisers’ widely correspond to the inhomogenous areas of the ARDS lung and can be present in up to 40% of the lung.

Although most of the literature on VILI concentrates on the possible dangers of tidal volume, mechanical ventilation in fact delivers mechanical power (i.e., energy per unit of time) to the lung parenchyma, which reacts to it according to its anatomical structure and pathophysiological status. The determinants of mechanical power are not only the tidal volume, but also respiratory rate, inspiratory flow, and positive end-expiratory pressure (PEEP). In the end, decreasing mechanical power, increasing lung homogeneity, and avoiding reaching the anatomical limits of the ‘baby lung’ should be the goals for safe ventilation in ARDS.

## Background

During the acute respiratory failure caused by inflammatory edema—the condition to which we will limit our discussion—the lungs present a high degree of inhomogeneity [1]. Indeed aerated, poorly aerated, and consolidated/collapsed regions do coexist throughout the lung parenchyma.

From the sternum to the vertebrae and in the supine position, the lung with acute inflammatory edema (acute respiratory distress syndrome (ARDS)) presents as a rough simplification: 1) few regions of possible hyperinflation (difficult to define by computed tomography (CT) due to the increased lung mass); 2) regions with normal ratio between gas and tissue (usually defined as well aerated); 3) regions with gas-tissue ratios lower than normal (usually defined as the ones with a ratio between gas and tissue below 1); and 4) completely gasless areas in the most dependent lung regions (situated at different lung heights depending on the severity of the syndrome). It is important to realize that these gasless regions may be due either to a complete collapse of ‘empty’ pulmonary units (which can be possibly reopened and refilled with gas) or to a complete consolidation of the pulmonary units, in which the inner space is occupied by solid/liquid material [2]. Obviously, the differences in lung inflation are a signal of inhomogeneity and we may infer that the difference in gas-tissue ratio (i.e., in inflation) between different lung regions may be due either to anatomical variations in a given area of interest, or to the presence of different forces acting on contiguous structures of the lung parenchyma.

The interest for the pathophysiology of the ARDS lung derives from the need (in most of these patients) for mechanical ventilation. This technique substitutes the respiratory muscles, completely or in part, in the role of providing the energy needed to inflate the lung. Therefore, the possible harm of mechanical ventilation derives from the interaction between the anatomical-physiological characteristics of the lung parenchyma and the mechanical power delivered to it. Ideally, a proper setting of mechanical ventilation should find the best compromise between mechanical power and lung structure. In extreme synthesis, we should provide the lowest mechanical power in a parenchyma made as much homogeneous as possible. In this brief paper we will give our view on the interaction between mechanical power and regional lung physiology.

## The lung parenchyma

### Forces acting on the lung

It is worth remembering that the pressure (i.e., force per surface unit) distending the lung is the transpulmonary pressure, which equals the difference between the pressure at the airway and the pleural pressure. The appropriateness of this terminology has been recently questioned [3]. In fact, in pure physiology, the airway opening pressure is the airway pressure measured at the beginning of the endotracheal tube, which may be split into two components: the one used to move gas through the airways (‘resistive’ component), and the one used to distend the lung (‘elastic’ component). Accordingly, the transpulmonary pressure, in this purely physiological view, is the difference between the airway opening pressure and the corresponding pleural pressure. The terminology used by intensivists (and some physiologists) is different, as it refers to the conditions in which the pressure at the airway opening is measured as the peak pressure (which includes the resistive and elastic components), the plateau pressure (assumed to be equal to the alveolar pressure when the flow is zero), and the end-expiratory pressure. The changes in esophageal pressure at peak, plateau, and positive end-expiratory pressure (PEEP) or zero end-expiratory pressure (ZEEP) would reflect the corresponding changes in the pleural pressure. The esophageal pressure is used as a surrogate of the pleural pressure, as its changes are equal to the changes of the pleural pressure. It must be noted that trying to equate the absolute esophageal pressure to the pleural pressure is more a physiological dream than a reality. We never found any association between absolute esophageal pressure and the anatomical characteristics of the lung after examining hundreds of CT scans in ARDS. In this paper, according to our previous work [4] and other authors [5, 6], we define the transpulmonary pressure as the difference between the airway pressure measured in static conditions (plateau and PEEP/ZEEP) and the corresponding difference in esophageal pressure:

$$ \varDelta {P}_L=\left( Pa{w}_{plat}- Pa{w}_{end-\mathit{\exp}}\right)-\left( Pe{s}_{plat}- Pe{s}_{end-\mathit{\exp}}\right) $$

where Paw_plat_ is the airway plateau pressure, Paw_end–exp_ is the airway pressure at PEEP or ZEEP, Pes_plat_ is the esophageal pressure at plateau and Pes_end–exp_ is the esophageal pressure at PEEP or ZEEP.

Accordingly, being:

$$ \frac{\varDelta {P}_L}{1\ L}={E}_L\kern0.5em \mathrm{and}\kern0.5em \frac{\varDelta {P}_{aw}}{1\ L}={E}_{rs} $$

it derives that:

$$ \varDelta {P}_L=\varDelta {P}_{aw}\ast \frac{E_L}{E_{rs}} $$

where ∆P_L_ is the driving transpulmonary pressure, ∆P_aw_ is the driving airway pressure, E_L_ is the lung elastance and E_rs_ is the total elastance of the respiratory system (i.e., E_rs_ = E_L_ + E_w_, where E_w_ is the chest wall elastance).

**Fig. 1:**
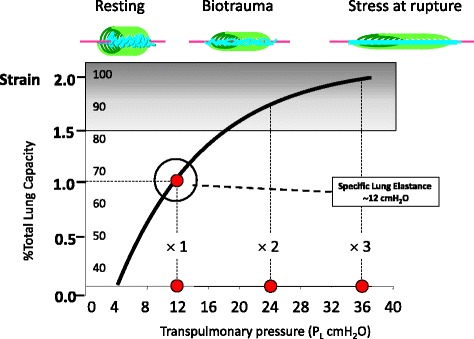
Strain (tidal volume divided by the functional residual capacity) and total lung capacity (*y* axis) are represented as a function of the applied transpulmonary pressure (*x* axis) [22]. In the resting position, the collagen fibers are folded within the elastic spring [23]. Increasing the transpulmonary pressure, the excessive strain may lead firstly to an inflammatory reaction and, when the collagen is completely unfolded, to stress at rupture. At ~ 12 cmH_2_O transpulmonary pressure (i.e., the specific elastance), the initial lung volume is doubled [7]

In a series of studies we found that the average E_L_/E_rs_ ratio in supine patients with ARDS was approximately 0.7 [7]. This indicates that at an airway plateau pressure of 30 cmH_2_O, the plateau transpulmonary pressure is approximately 21 cmH_2_O. However, we found that in single individuals the E_L_/E_rs_ ratio may vary from 0.2 to 0.8 [7]. This indicates that, at the plateau pressure usually accepted as the threshold for a ‘safe’ mechanical ventilation, the transpulmonary pressure may be as low as 6 cmH_2_O (with all the risks of hypoventilation and/or collapse) and as high as 24 cmH_2_O, fully within the borders of the total lung capacity (TLC). In experimental models we found that lethal ventilation occurs when the total lung capacity region is reached [8]. In Fig. 1 we report the classical volume-transpulmonary pressure curve, readapted to the scenario of ventilator-induced lung injury (VILI). As shown, whatever the initial lung size (that we call ‘baby lung’ in ARDS), its inspiratory capacity is reached at 2.5–3 times the initial volume. Indeed, in a ‘baby lung’ of 300 mL, the TLC is reached at a volume of 750–900 mL. If the delivered tidal volume is in this order of magnitude, it would generate a transpulmonary pressure (also known as lung stress) of ~24 cmH_2_O and a lung strain (i.e., the ratio of tidal volume to the functional residual capacity (FRC)) of 2.5–3.0, which have been shown to be lethal in animal models. Therefore, the lung size is the first factor to be considered for the development of VILI.

### The stress raisers

Although in an extremely small ‘baby lung’ it is possible to reach the TLC with the tidal volume (TV), this mechanism cannot explain the harm observed in human ARDS with a tidal volume of 12 mL/kg [9, 10]. Indeed, reaching the TLC in most of the ARDS patients would require a tidal volume greater than 12 mL/kg. A possible explanation of the damage observed in ARDS patients ventilated with 12 mL/kg tidal volume compared to 6 mL/kg is the presence of local factors which may locally multiply the applied pressures, with consequent increase in local stress and strain. This may occur to a greater extent with greater lung inhomogeneity, according to a theoretical model developed by Mead in the 1970s [11] and popularized by Lachmann in the 1990s [12]. Accordingly, if we imagine a given 10-kg load, hanging on 10 elastic fibers, each fiber will carry 1 kg. If for any reason (as an example, atelectasis) one fiber does not carry its own fraction of load anymore, the remaining fibers will carry ~ 1.11 kg each. If the inhomogeneity extends further to four fibers, the remaining six will carry ~ 1.67 kg each, and so on. The load in our case is represented by the transpulmonary pressure recorded at plateau. In a theoretical computation, and referring to a more complex geometrical model, Mead found that the multiplication factor for pressure at the interface between completely collapsed (volume = 1) and completely distended pulmonary units (volume = 10) would be equal to [11]:

$$ {\left(\frac{10}{1}\right)}^{\frac{2}{3}}=4.64 $$

from which it has been often claimed that at 30 cmH_2_O the local pressure could be as high as ~ 120 cmH_2_O [12]. Actually, when we estimated the inhomogeneity by comparing the inflation ratio of neighboring lung regions [1], we found that the multiplication factor was ~ 2. According to the ARDS severity, the stress raisers were present in up to 40% of the lung parenchyma, suggesting that a given transpulmonary pressure is doubled in ~ 40% of the lung.

**Fig. 2:**
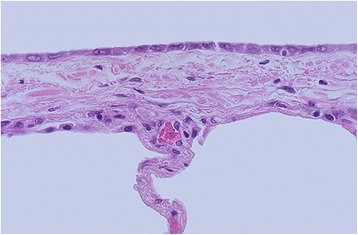
Visceral pleura from which an alveolar wall departs. The interface between these two structures of different elasticity acts as a stress raiser with a possible local multiplication of stress and strain. Photograph courtesy of Dr. Edward C. Klatt, M.D., © WebPath

**Fig. 3:**
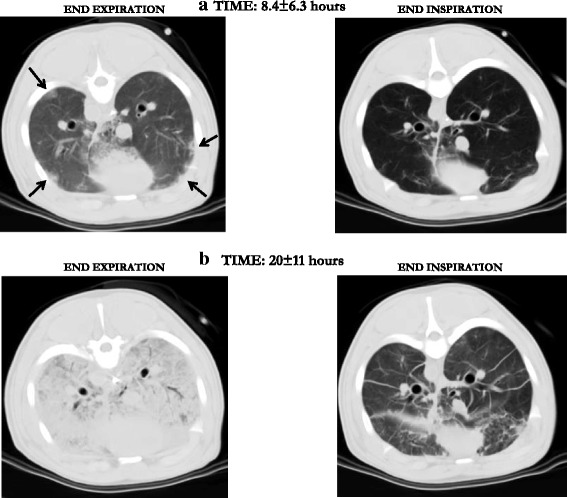
**a** The first button-like densities (*arrows*) appear at the interface with the visceral pleura and, after 20 h of 2.5 strain ventilation (**b**), are extended to the whole parenchyma. Note that these VILI lesions are almost fully recruitable, suggesting that they develop primarily as interstitial edema

As a proof-of-concept of the presence of the stress raisers we hypothesized that the lesions during mechanical ventilation would firstly occur at the interfaces between regions of different elasticity, which, in healthy lungs, are mostly represented by the interfaces between the visceral pleura and the subpleural alveoli (see Fig. 2). Actually, we found that after an average of 8 h of mechanical ventilation small lesions start to occur at the pleuric-alveolar interfaces, and extend in about 20 h to the whole lung (see Fig. 3) [13]. Therefore, the bulk of data available strongly suggest that there is an indication to reduce the lung inhomogeneity as much as possible in moderate-severe and severe ARDS patients through appropriate maneuvers (essentially prone positioning).

## The mechanical power

The literature on VILI concentrates primarily on the possible danger of tidal volume. Recently the possible relevance of airway driving pressure (i.e., tidal volume normalized to the respiratory system compliance) has been emphasized [14]. Other possible causes of VILI have been identified in the respiratory rate [15] and in the inspiratory flow [16]. Additional factors, such as total/regional perfusion, local acidity, and temperature, may play a role in modulation of VILI but, for simplicity, will not be considered here.

**Fig. 4:**
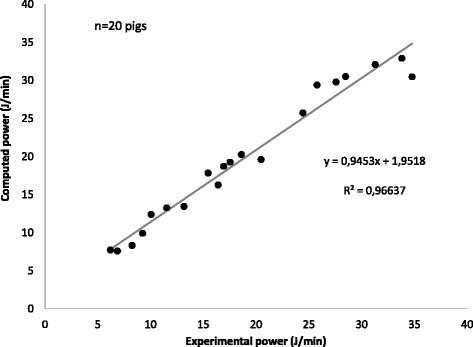
Relationship between the power computed through the power equation and that measured through the graphic analysis of the pressure-volume loops. As shown, the relationship (*p* < 0.001) is close to the identity line

In a series of experiments in pigs, aiming to identify a possible threshold for VILI, we found that the VILI was a function of how a harmful strain of 2.5 was reached, which is different to that observed by Dreyfuss et al. in rats [17]. In fact, while in the rats the VILI rapidly occurred at certain plateau pressures, independently of the way through which the plateau was reached (i.e., with or without PEEP), we found that ventilation at 15 bpm respiratory rate and strain equal to 2.5 (i.e., total volume close to 2.5 times the FRC) was lethal if provided totally as tidal volume and completely innocent (without any damage) if 75% of the added volume was provided as PEEP and 25% as tidal volume [18]. This led us to hypothesize that the damage was not due to the tidal volume per se, but to the product of tidal volume and pressure. This product (i.e., absolute pressure multiplied by the tidal volume) is the tidal energy delivered to the lung parenchyma. Actually, if a given amount of energy is given at a different rate, the mechanical power delivered per minute may be completely different. As a proof of concept, we ventilated pigs with a 2.5 strain (which is lethal when delivered 15 times per minute) at the rate of 3, 6, 9, 12, and 15 bpm and we found that below certain levels of mechanical power no VILI occurred after 54 h of mechanical ventilation [19]. Therefore, starting from the equation of motion of the respiratory system, we developed the power equation [20] simply by multiplying each component of the original equation by the change in volume and the respiratory rate. We found an impressive relationship in humans between computed and measured mechanical power, as well as in experimental animals (see Fig. 4). Considering the effects of the single components of mechanical power, we found that doubling the tidal volume or the driving airway pressure (i.e., plateau minus PEEP) leads to a fourfold increase in mechanical power (exponent 2). In contrast, doubling the respiratory rate led to an increase in mechanical power of ~ 2.5 times (exponent 1.4) and of two times (exponent 1) if the PEEP is doubled [20].

This ‘mechanical hypothesis’ obviously needs further studies: 1) the mechanical power should be related to the transpulmonary pressure; and 2) it should be normalized for lung size and, likely, for specific lung elastance to allow comparison between different mammalian species. It is possible that in identifying an unsafe threshold for mechanical ventilation, based on ‘lung-directed/normalized’ mechanical power, a more rational approach to safe mechanical ventilation and indications for possible extracorporeal support may be established.

## Conclusion

Mechanical ventilation is applied to the ventilatable fraction of the ARDS lung (the ‘baby lung’). The anatomical threshold is likely represented by the total lung capacity which may be reached through local pressure rises depending on the lung inhomogeneity. Inside this framework, we can consider that:

- Whatever the decrease in mechanical power (due to the reduction of whichever of its components) should decrease the likelihood of ventilator-induced lung injury.
- The best available maneuver to increase lung homogeneity (without causing any increase in mechanical power) is prone positioning [21]. This is clearly indicated in patients with moderate-severe and severe ARDS, who present with the highest degree of lung inhomogeneity.
- PEEP has a dual effect: on one side, it may decrease lung inhomogeneity, at least in the patients in whom lung collapse can be substantially reduced. On the other hand, for a given tidal volume, PEEP increases the mechanical power and the likelihood of reaching the anatomical threshold for VILI, i.e., the total lung capacity.
